# Association of nuts and unhealthy snacks with subclinical atherosclerosis among children and adolescents with overweight and obesity

**DOI:** 10.1186/s12986-019-0350-y

**Published:** 2019-04-08

**Authors:** Maryam Aghayan, Golaleh Asghari, Emad Yuzbashian, Pooneh Dehghan, Hossein Khadem Haghighian, Parvin Mirmiran, Maryam Javadi

**Affiliations:** 1 Department of Nutrition, School of Health, Qazvin University of Medical Sciences, Qazvin, Iran; 2 Department of Clinical Nutrition and Dietetics, Faculty of Nutrition Sciences and Food Technology, National Nutrition and Food Technology Research Institute, Shahid Beheshti University of Medical Sciences, Tehran, Iran; 3 Nutrition and Endocrine Research Center, Research Institute for Endocrine Sciences, Shahid Beheshti University of Medical Sciences, P.O. Box: 19395-4763, Tehran, Iran; 4 Department of Imaging, Research Development Center, Taleghani Hospital, Shahid Beheshti University of Medical Sciences, Tehran, Iran; 5 Children Growth Research Center, Qazvin University of Medical Sciences, P.O. Box: 34159-14595, Qazvin, Iran

**Keywords:** Nuts, Snacks, Substitution, cIMT, Atherosclerosis

## Abstract

**Background:**

The process of atherosclerosis begins early in childhood and usually remains asymptomatic until later in life. Carotid intima-media thickness (cIMT) as a marker of subclinical atherosclerosis could identify early vascular alterations. Unhealthy snacks consumption is associated with obesity and other CVD risk factors in children and adolescents. The aim of this study is to investigate the association of different snack substitution and cIMT among overweight and obesity children and adolescents.

**Methods:**

A total of 339 participants aged 6 to 13 years with the body mass index Z score ≥ 1 based on WHO criteria enrolled in this study. We measured food intakes of participants by validate and reliable food frequency questionnaire (FFQ). Carotid intima media thickness was measured in the common carotid artery with high-resolution ultrasonography.

**Results:**

After controlling for confounders, intake of nuts had a negative relationship with cIMT (β = 0.135 mm *P* value = 0.009). Moreover, participants in the last tertile of nuts intake had 59% lower risk of high cIMT in comparison with those who consumed less than 0.64 serving/wk./1000Kcal of nuts (P for trend = 0.010). Substituted of nuts intake with sweet unhealthy snacks had a negative relationship with cIMT (β = 0.15 mm). There was no other significant association between energy-dense nutrient-poor solid snacks and cIMT.

**Conclusions:**

Our findings emphasize the impact of nuts consumption as a healthy snack on subclinical stages atherosclerosis. Clinical trial studies could examine the effect of different kinds of nuts consumption on cIMT and complications of CVD risk factors.

**Electronic supplementary material:**

The online version of this article (10.1186/s12986-019-0350-y) contains supplementary material, which is available to authorized users.

## Background

The process of atherosclerosis, the main cause of cardiovascular disease (CVD), begins early in childhood and usually remains asymptomatic until later in life [1]. To prevent the harmful consequences of CVD, it is important to determine high-risk individuals at an early age [2]. Measuring carotid intima-media thickness (cIMT), a noninvasive, feasible, and reliable approach, in children and adolescents with obesity has been recommended by the Association for European Pediatric Cardiology (AEPC), as it is capable of identifying early vascular alterations and thereby preventing myocardial infarction and stroke in adulthood [3].

The etiology of CVD is defined by the interaction of both genetic and environmental factors [4]. One potentially important modifiable environmental factor is snack consumption. It has become major part of daily energy intake, particularly among Iranian children which meet 40% of their total energy needs through snack consumption [5].

There are two different types of snacks, based on the content of nutrients, including energy-dense nutrient-poor (e.g., potato chips, candy, cake) and nutrient-dense (e.g., unsalted nuts) snacks [6]. Consumption of unhealthy snacks, rich in trans fatty acids, sodium, and simple sugars disturb the lipid and glucose metabolism and are related to the CVD risk factors [7]. However, the anti-inflammation and antioxidant compounds that are common in nuts as a healthy snack, make them a valuable substitution for unhealthy snacks and link them to the reduction of CVD risk factors [8].

According to previous studies, substitution of desirable dietary fatty acids for other macronutrients decreased CVD risk factors among adult population [9, 10]. However, the association of nuts substitution for unhealthy snacks on cIMT as subclinical atherosclerosis among children and adolescents has not yet been studied. Since children and adolescents with excess weight have more snack consumption and childhood obesity is related to adulthood atherosclerosis [11, 12], this study was designed to determine the relationship between nut consumption and cIMT among participants, and a specific aim was to examine the relation of nuts replacement by using substitution analysis for unhealthy snacks (salty and sweet) on cIMT.

## Methods

### Study population

For this cross-sectional study, 378 overweight and obese children (age- and sex-specific body mass index (BMI) Z-score ≥ 1 based on the criteria established by the World Health Organization) aged 6–13 years, were recruited from three main districts of Tehran, the capital of Iran. Individuals were eligible for inclusion if they had no known medical illnesses such as diabetes, liver, or kidney diseases, were not taking any dietary supplements, or using pharmaceutical agents that affect glucose and lipid metabolism. Twenty-three participants who missed anthropometric, dietary, biochemical and ultrasound assessments were excluded. Furthermore, those who over- or under-reported were also excluded (*n* = 16). To define over- and underreports, the reported energy intake was divided by the estimated energy requirement (EER), calculated according to equations proposed by the Institute of Medicine who were not within the 2SD range [13]. Finally, the statistical analysis was performed on 339 overweight and obesity children and adolescents.

The design of this study was approved by the institutional ethics committee of the Research Institute for Endocrine Sciences, affiliated to the Shahid Beheshti University of Medical Sciences, and written informed consent was obtained from participants’ parents.

### Measurements

Anthropometric measurements were taken by a trained nutritionist according to standard methods. Weight was measured to the nearest 100 g, while participants were minimally clothed without shoes, using the scale function of the GAIA 359 PLUS (Jawon Medical Co. Ltd., Shinsang, Korea). Height was measured to the nearest 0.5 cm, in standing position with shoulders in normal alignment and without shoes, using a standard tape. BMI was calculated as weight divided by the square of the height (kg/m^2^). Waist circumference was measured to the nearest 0.5 cm using a measuring tape in the standing position after a gentle respiration, at the level of the umbilicus.

Blood pressure of participants was measured after 15 min of resting with a standard mercury sphygmomanometer on the right arm. It was measured twice and the mean of the two measurements was recorded as the participant’s blood pressure. Pubertal status was classified according to the definitions of Tanner stages, determined by a well-trained endocrinologist. The pubertal developmental stage was categorized into 2 groups based on breast and genital stages (pre-pubertal: boys at genital stage I, girls at breast stage I; pubertal: boys at genital stage ≥II, girls at breast stage ≥II).

Physical activity was assessed by using the Modifiable Activity Questionnaire (MAQ), to calculate metabolic equivalent task minutes per week. Reliability and moderate validity have been specified previously for the Persian translated MAQ in adolescents [14]. Low level of physical activity were considered as metabolic equivalent task < 600 min/wk.

Blood samples were drawn between 7:00 and 9:00 AM from all study participants after 12–14 h of overnight fasting. All the blood analyses were done at the Tehran Lipid Glucose Study (TLGS) research laboratory on the day of blood collection. Fasting plasma glucose (FPG) was measured by the enzymatic colorimetric method using glucose oxidase. Serum triglycerides (TGs) were assayed using an enzymatic colorimetric method with glycerol phosphate oxidase. These analyses were performed using commercial kits (Pars Azmoon, Tehran, Iran) and a Selectra 2 autoanalyzer (Vital Scientific, Spankeren, The Netherlands), with intra- and inter-assay coefficients of variation (CVs) of 1.1 and 1.4% for FPG and both less than 2% for TGs, respectively.

### Dietary assessment

Dietary intake was gathered using a reliable and validated, semi-quantitative food frequency questionnaire (FFQ) to assess the regular dietary intakes of participants over the previous year. Trained dieticians, during face-to-face interviews, asked participants and their mothers (when children were unable to recall) to designate their intake frequency for each food item consumed during the past year on a daily, weekly, or monthly basis. For each food item on the FFQ, a portion size was specified using US Department of Agriculture (USDA) serving sizes (eg, bread, 1 slice; apple, 1 medium; dairy, 1 cup) whenever possible; if this was not possible, household measures (eg, beans, 1 tablespoon; chicken meat, 1 leg, breast, or wing; rice, 1 large, medium, or small plate) were chosen and were then converted to grams and servings. Energy and nutrient contents were obtained from USDA food composition tables (FCT) because Iranian FCTs are incomplete and with limited data on nutrient content of raw foods and beverages foods, although, Iranian FCTs were used for traditional food items (Like Kashk) that are not listed in the USDA FCT. To evaluate the reproducibility of the FFQ, 132 participants completed the questionnaire twice, with a 14-month interval. Twelve dietary recalls were also collected (1 per month) to assess the validity of the FFQ. The adjusted correlation coefficients to assess validity of the FFQ for total food groups was 0.44 (nuts: 0.54) in men and 0.37 (nuts: 0.39) in women; intraclass correlation coefficients, which reflect the reproducibility of food groups in the FFQ, was 0.51 in men (nuts: 0.34) and 0.59 in women (nuts: 0.52) [15].

Snacking patterns are usually classified based on two main criteria; the type of snack and the time when the snack was eaten [16]. In the current study, snacks were divided into low-energy high-nutrient (nuts) and unhealthy energy-dense nutrient-poor solid foods (salty and sweet). Sweet snacks included candies, chocolates, cookies, cakes, biscuits, confectionery, caramels, and traditional Iranian confectioneries, Gaz, Sohan, Noghl, Halva, Yazdi cakes, and salty ones included potato chips and puff (a corn snack or crisp coated with a mixture of cheese or cheese flavored). Total energy dense nutrient poor solid snacks were calculated by the summation of the sweet and salty snacks. Moreover, nuts included all kinds of tree nuts and seeds, including peanuts, almonds, walnuts, pistachios, hazelnuts, and seeds. Sugar-sweetened beverages or high nutritional value snacks such as fruits, vegetables, and dairy products were not included in this study.

### Carotid intima-media thickness measurement

Carotid intima-media thickness was measured by a trained radiologist (P.D.). Participants were examined in the supine position with head slightly extended and rotated to the opposite side of examination; carotid arteries were investigated using a high-resolution Samsung ultrasound machine (model UGEO WS80A) with a linear-array transducer operating at a frequency of at least 7 MHz. Depth, gain and focus was adjusted for each participant individually so that the arterial lumen was completely anechoic and in the center of the image. Common cIMT was measured from longitudinal B-mode images of the distal 1 cm of the far wall of each common carotid artery (CCA) between the intimal-luminal and the medial-adventitial interfaces of the carotid artery wall represented as a double-line density on the ultrasound image. Measurements were performed using the automated edge-tracking software (automated IMT calculator) which obviated the need to perform manual measurements [1].

### Statistical analysis

Statistical analysis was performed using the Statistical Package for Social Sciences (version 15.0; SPSS, Chicago IL). The normality of the distribution of variables was assessed by the Kolmogorov-Smirnov tests. Characteristics of participants according to tertiles of nuts intake were expressed as mean ± SD or median and interquartile range for continuous and percentages for categorical variables. To investigate the trend of variables according to the tertiles, ANOVA and chi-square test were used for continuous and categorical variables, respectively. Plasma TG, systolic and diastolic blood pressure were skewed, so the log transformation was used. A linear regression model was used to assess the relation of cIMT with snack consumptions. Multivariable logistic regression models were used to examine the association of snack consumptions with cIMT. The odds ratio (OR) and 95% confidence intervals (CIs) for the incidence of high cIMT were calculated. Because of the continuous nature of cIMT, we binned it into tertiles and put the two first tertiles as low cIMT and the last one as high cIMT. The first model was adjusted for age, sex, total energy intake, physical activity and pubertal status and the second was further adjusted for BMI.

We estimated the associations of substituting 1 serving of nuts for 1 serving of sweet, salty, and energy-dense nutrient-poor solid snacks with cIMT by including them as continuous variables in the same multivariate model, which also contained non-dietary covariates and total energy intake. The difference in their coefficients and in their own variances and covariance were used to estimate the substitution associations [17].

In this study, we have conducted the sensitivity analysis to assess the robustness of the results. For this reason, we have added more atherosclerosis risk factor such as systolic (SBP) and diastolic (DBP) blood pressure, and intakes of saturated (SFA), monounsaturated (MUFA), and polyunsaturated (PUFA) fatty acids as further potential confounders.

In addition, we used Spline regression to examine a dose-response relation between snacks consumption and risk of high cIMT.

## Results

Mean (±SD) age and BMI z-score of participants were 9.3 ± 1.7 years and 2.5 ± 0.7 respectively. The obesity and low physical activity of children and adolescents were 68.4 and 56.3%, respectively and 82% had started puberty. The mean (±SD) intake of nuts, sweet, and salty snacks were 1.8 ± 2.5, 3.0 ± 1.8 and 1.0 ± 1.2 servings/wk./1000Kcal, respectively.

There was no significant difference between baseline demographics and characteristics of participants across categories of nuts intake (Table 1). Mean dietary intakes of participants across tertiles of nuts consumption are shown in Table 2. By increasing the tertiles of nuts consumption, percent of energy from total fat, monounsaturated fatty acid (MUFA), and polyunsaturated fatty acid (PUFA) increased. Participants in the highest compared with the lowest tertile of nuts had lower intakes of carbohydrates and fiber.

**Table 1:** Baseline characteristic of children and adolescents according to tertiles of nut consumption

	Nuts (serving/wk./1000Kcal)			P for trend
	T1 (< 0.64)	T2 (0.65–1.60)	T3 (> 1.61)	
Participants (n)	113	113	113	
Median (serving/wk./1000Kcal)	0.34	0.99	2.78	
Age (years)	9.2 ± 1.7	9.3 ± 1.7	9.4 ± 1.8	0.471
Girls (%)	44.6	44.2	54.4	0.142
WC (cm)	80.5 ± 10.6	80.6 ± 8.4	80.9 ± 9.7	0.707
BMI (kg/m2)	23.4 ± 3.5	23.2 ± 2.9	23.3 ± 3.3	0.864
BMI z-score	0.2 ± 1.0	0.1 ± 0.9	0.1 ± 1.0	0.864
Obesity (%)	68.8	69.9	66.7	0.735
Low physical activity (%)	61.6	52.2	55.3	0.606
Pubertal (%)	80.4	78.8	88.6	0.101
SBP (mmHg)	105.1 ± 12.8	104.2 ± 13.0	105.3 ± 13.7	0.737
DBP (mmHg)	64.8 ± 8.4	65.2 ± 8.4	66.2 ± 10.5	0.251
High blood pressure (%)	19.6	25.7	25.4	0.307
FPG (mg/dl)	90.8 ± 9.5	91.2 ± 9.0	89.8 ± 8.9	0.341
High FPG (%)	19.6	14.3	15.9	0.456
Serum triglycerides (mg/dl)	107.5 (80.0–134.0)	101.0 (74.0–134.0)	106.0 (75–152.2)	0.195
High triglyceride (%)	44.6	42.1	47.8	0.635

*WC* waist circumference, *BMI z-score* body mass index z-score, *SBP* systolic blood pressure, *DBP* diastolic blood pressure, *FPG* fasting plasma glucose

Data are represented as mean ± SD or median (IQR 25–75) for continuous variables and as percentages for categorical variables

ANOVA and chi-square test were used for continuous and categorical variables, respectively

**Table 2:** Dietary Factors of children and adolescents according to tertiles of nut consumptions

	Nuts (serving/wk./1000Kcal)			P for trend
	T1 (< 0.64)	T2 (0.65–1.60)	T3 (> 1.61)	
Total energy intake (Kcal)	2834	2911	2809	0.664
Carbohydrate (% energy)	56.8 ± 5.6	56.8 ± 5.6	54.4 ± 5.6	0.000
Dietary fiber (g/1000Kcal)	18.7 ± 6.1	17.0 ± 6.1	16.6 ± 5.3	0.047
Total fat (%energy)	31.9 ± 5.6	32.5 ± 5.3	34.6 ± 5.1	0.000
SFA (%energy)	10.1 ± 2.5	10.1 ± 2.3	10.5 ± 2.2	0.106
MUFA (%energy)	10.1 ± 2.3	10.2 ± 2.2	10.8 ± 2.2	0.010
PUFA (%energy)	6.6 ± 2.1	6.7 ± 1.9	7.6 ± 1.8	0.000
Protein (%energy)	13.4 ± 2.1	13.0 ± 2.1	13.6 ± 2.	0.265
Vegetable (serving/wk./1000Kcal)	5.3 ± 2.7	6.0 ± 3.3	6.2 ± 3.7	0.089
Fruit (serving/wk./1000Kcal)	6.7 ± 3.4	7.9 ± 4.7	7.6 ± 3.7	0.436
Sweet snacks (serving/wk./1000Kcal)	2.8 ± 1.4	3.2 ± 1.9	3.0 ± 1.9	0.745
Salty snacks (serving/wk./1000Kcal)	1.0 ± 1.3	1.0 ± 1.3	1.0 ± 0.9	0.938
Energy-dense nutrient-poor solid snacks (serving/wk./1000Kcal)	3.9 ± 1.7	4.2 ± 2.3	4.5 ± 2.0	0.811

*SFA* saturated fatty acid, *MUFA* monounsaturated fatty acid, *PUFA* polyunsaturated fatty acid

Data are represented as mean ± SD

Linear regression was used

Standardized coefficient of cIMT and snack consumptions are presented in Table 3. There was a negative relation between nut consumptions and cIMT after controlling for sex, age, energy intake, pubertal status, and physical activity (*β* = − 0.135, *P* = 0.009). This relation remained significant after further adjustment for BMI. However, there was no significant association between intake of energy-dense nutrient-poor solid snacks and cIMT, in the fully adjusted model.

**Table 3:** Standardized coefficients of snack consumption with cIMT in children and adolescents

	carotid intima-media thickness	
	β	*P* value
Nuts (serving/wk./1000 kcal)		
Model1	−0.106	0.051
Model2	−0.135	0.009
Model3	−0.135	0.009
Energy-dense nutrient-poor solid snacks (serving/wk./1000 kcal)		
Model1	−0.057	0.293
Model2	−0.008	0.874
Model3	−0.008	0.882

Model 1 was crude

Model 2 was adjusted for sex, age, energy intake, pubertal status, physical activity

Model 3 was adjusted for model 2 variables plus BMI

Linear regression was used

The risk of high cIMT according to tertiles of snack consumptions is presented in Table 4. In comparison with the participants in the lowest tertile of nuts intake, those in the highest one had an indirect relation with risk of high cIMT in the fully adjusted model (OR: 0.41; 95% CI: 0.21 to 0.79, *P* for trend = 0.010). However, there was no relationship between consumption of energy-dense nutrient-poor solid snacks and risk of high cIMT even after adjustments for potential confounders.

**Table 4:** Multivariable-adjusted ORs (95% CIs) for the prevalence of high cIMT by tertiles of snack consumption in children and adolescents

	Intakes			P for trend
	T1 (*n* = 112)	T2 (*n* = 114)	T3 (*n* = 113)	
Median nuts (serving/wk./1000 kcal)	0.34	0.99	2.78	
Model 1	1.00	0.83 (0.47–1.46)	0.49 (0.27–0.89)	0.018
Model 2	1.00	0.72 (0.39–1.31)	0.42 (0.22–0.81)	0.011
Model 3	1.00	0.70 (0.38–1.28)	0.41 (0.21–0.79)	0.010
Median energy-dense nutrient-poor solid snacks (serving/wk./1000 kcal)	2.13	3.83	5.83	
Model 1	1.00	0.95 (0.54–1.69)	0.76 (0.42–1.37)	0.367
Model 2	1.00	1.15 (0.62–2.13)	1.02 (0.54–1.91)	0.954
Model 3	1.00	1.13 (0.61–2.11)	1.00 (0.53–1.89)	0.985

*cIMT* carotid intima-media thickness

Model 1 was crude

Model 2 was adjusted for sex, age, energy intake, pubertal status, physical activity

Model 3 was adjusted for model 2 variables plus BMI

Using the median values for tertiles of snacks as continuous variables

**Fig. 1:**
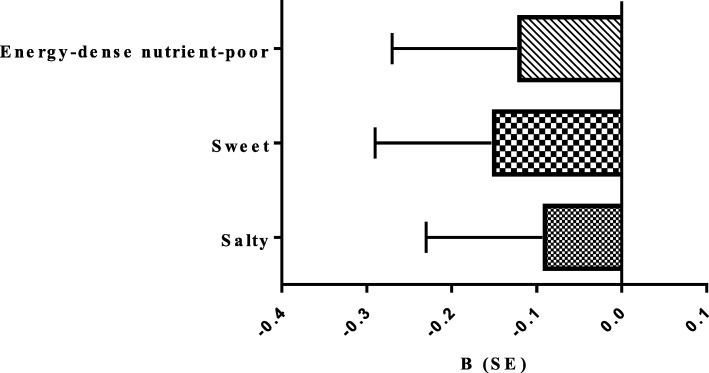
Substituting one serving of nuts for one serving of salty, sweet and total energy-dense nutrient-poor solid snacks and cIMT. Substitution of one serving of nuts for one serving of sweet snacks decreased cIMT.cIMT; carotid intima media thickness

**Fig. 2:**
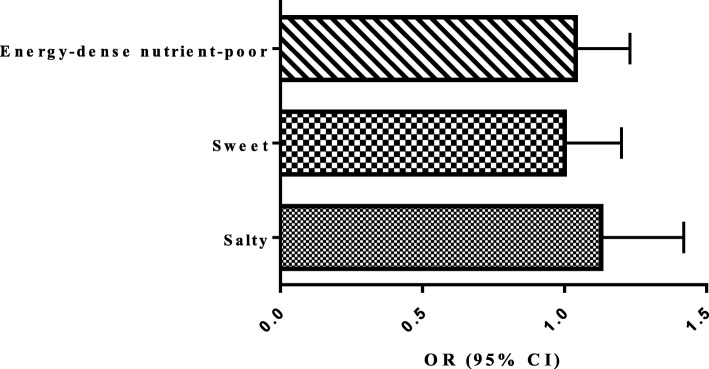
Substituting one serving of nuts for one serving of salty, sweet and total energy-dense nutrient-poor solid snacks and risk of high cIMT. Substitution of one serving of nuts for one serving of sweet, salty and total energy-dense nutrient-poor solid snacks did not change the risk of high cIMT. cIMT; carotid intima media thickness. OR; odds ratio

Substitution analysis for estimating the replacement of one serving of nuts as a healthy snack with sweet, salty and total energy-dense nutrient-poor solid snacks is shown in Figs. 1 and 2. Substituting one serving of nuts for one serving of sweet but not salty and total energy-dense nutrient-poor solid snacks had inverse relationship with the cIMT (β = − 0.15, 95% CI = -0.29 to − 0.00). Moreover substitution of nuts for sweet, salty and energy-dense nutrient-poor solid snacks was not in relationship with risk of high cIMT in the fully adjusted model.

**Fig. 3:**
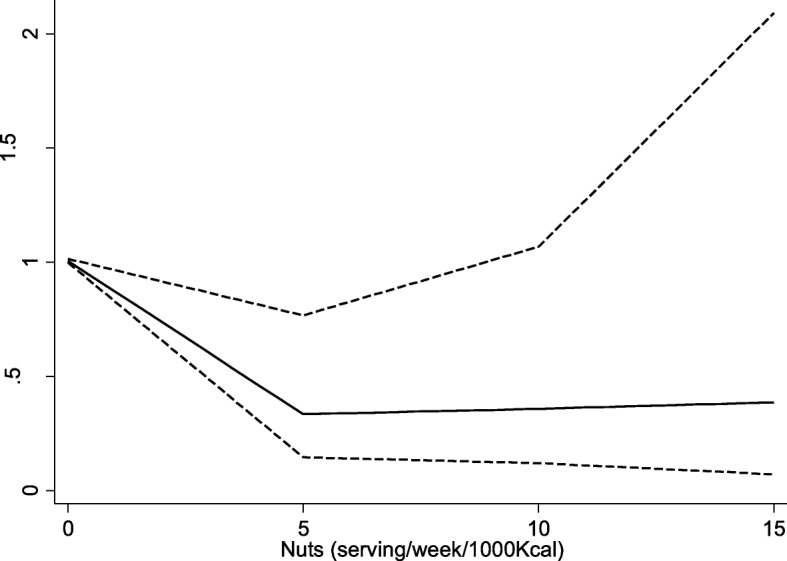
dose-response relationship between nuts intake and risk of high cIMT. Brocken lines represent 95% CI

As Fig. 3 shows, there was a negative linear relationship between nuts intake and risk of high cIMT; however, this relationship became plateau after the point where nuts consumption was more than 5 serving/wk./1000Kcal.

In the sensitivity analysis, the association of nuts intake and unhealthy snacks with cIMT and the risk of high cIMT did not substantially change when we further adjusted for SBP, DBP, SFA, MUFA, and PUFA (data was shown on Additional files 1: Tables S1 and S2).

## Discussion

In this cross-sectional study, nuts intake was inversely associated with the cIMT, independent of several known confounders. Compared with participants in the first tertile of nuts intake, those who were in the highest tertile had a 59% lower risk of high cIMT. Moreover, substitution of one serving of nuts for one serving of sweet snacks had a negative relationship with cIMT.

To the best of our knowledge, this is the first study that has investigated the relationship between nut consumptions and cIMT and also the association of nuts substitution for unhealthy snacks with cIMT among children and adolescents. However, a number of observational studies have investigated the relationship between snack consumption and CVD risk factors [18, 19]. Results of the National Health and Nutrition Examination Survey (NHANES) study indicated that consumption 7 g/day of nuts was related to decreased diastolic blood pressure among adolescents [18]. Identification and prevention of dietary and lifestyle-induced health effects in children and infants (IDEFICS) study, which investigated the relation of food intakes and CVD risk score among children aged 2–9 years, revealed that nuts and seed intakes decreased the risk of CVD score by 38% in boys [19]. Moreover, the desirable effects of nuts in the frame work of the Mediterranean diet on cIMT has been confirmed in two other studies [20, 21]. According to the clinical trial study of Gianini et.al, the Mediterranean diet decreased cIMT by 0.03 mm and improved lipid profiles in pre-pubertal hyper-cholesterolemic children with mean age of 7.5 years [20]. In a recent systematic review by Peterson et.al, results indicated that Mediterranean style dietary patterns may reduce cIMT progression in all age groups [21]. However, it is important to consider the synergistic effect of nuts and other healthy food items as components of the Mediterranean diet on CVD risk factors. Comparison of our findings with others is difficult, due to the very limited number of studies as well as different study designs, sample sizes, and use of different variables; nevertheless, based on all their results, nuts intake reduced cIMT and other CVD risk factors among children and adults.

To date, no other studies have investigated the relation of unhealthy snack consumption and subclinical atherosclerosis among children and adolescents. However, some limited controversial findings investigated the association of unhealthy snacks and CVD risk factors with metabolic syndrome [19, 22, 23]. Our previous study showed consumption of unhealthy snacks increased the risk of the incident metabolic syndrome and hypertension [22], findings in contrast to those of a cross-sectional study performed in the United States, that candy consumption did not have a relationship with blood pressure and lipid profiles of both children and adolescents [23], which is in line with results of the IDEFICS study which found that sweets decreased the CVD risk score by 48% and had a protective effect on CVD risk factors [19]. The relation of dietary patterns and cIMT has also been studied. The Cardiovascular Risk in Young Finns study, (subjects aged 3–18 years) investigated the relation of childhood dietary patterns on adulthood CVD risk factors and progression of atherosclerosis by measuring cIMT [24, 25]. Mikkila et.al showed that the more adherence to the traditional dietary pattern characterized by high consumption of potatoes, butter, sausages, and coffee was associated with cIMT, although one SD increase in scores of the healthy dietary pattern (characterized by high consumption of vegetables, legumes and nuts, tea, and cheese) did not have any relation to cIMT in both genders [25]. In the current study, we found no relationship between intakes of energy-dense nutrient-poor solid snacks and cIMT. Carotid intima media thickness as structural progression of the vascular wall thickening takes time to show its atherosclerosis effects. Moreover, we observed a very narrow range of cIMT among participants in young age group and in newly pubertal status. Besides, the cross-sectional design of the present study makes the observation of this relationship quite complicated.

An interesting point of current study was using the substitution model of different snack types; which indicated that cIMT decreased when nuts replaced for sweet unhealthy snacks; supporting the protective effect of nuts in the single dietary model. Replacing sweet unhealthy snacks with nuts is important in this regard that according to Karimian et.al, Iranian students received 40% of their total energy needs from snack consumption which provides a big part of their total energy intake [5], and replacement led to higher intake of nutrient-rich diets that are lower in energy-dense nutrient-poor food items and increase intakes of more favorable fatty acids [26]. Unhealthy diets in childhood can deteriorate vascular changes and increased risk of CVD in adulthood. Despite the fact that cIMT reduction which observed in the current study was very small, this small change is very important due to its effects on several CVD events in adulthood [27].

The beneficial effects of nuts may be described by their unique nutrient profiles including fiber, vitamins E, potassium, magnesium, antioxidants, polyphenols, desirable fatty acid profiles, through decrease of total LDL-cholesterol, reduced lipid peroxidation, improved endothelial function, thereby having a beneficial effects on cardiovascular risk factors [8, 28, 29]. Analyzing the childhood serum cholesterol ester fatty acid as markers of dietary fatty acid intake, childhood saturated fatty acid was directly, and omega 6 fatty acids were inversly associated with adulthood cIMT [30]. We examined the thickness of arterial wall as a predictor of the subclinical stage of endothelial dysfunction. Agustench et.al indicated that nut consumption has beneficial effects on endothelial function, inflammatory markers, and insulin resistance that are related to sufficient bioactive compounds that are common in nuts [31].

Our study has some limitations. First, although the statistical models for various confounders were carefully controlled, it is possible to miss the exclusion of unknown residual confounders. Second, because of the cross-sectional design of the study, we could not assess the causality of study. Third, according to Vance et.al, the underreporting of unhealthy food items among children with overweight and obesity is much higher, which made our interpretation difficult [32]. However, with the help of expert interviewers and taking information from mothers about food items minimizes recall limitations. Moreover, we excluded the under and over reported. Fourth, in this study, we could not define the time and place of snacking and the amount of energy intake in each snack type, which could have helped us with better interpretation. Another limitation was that liquid snacks including milk and sugar-sweetened beverages were not considered in the analysis. Moreover, there is no data regarding the validity of cIMT, however, in this study it was measured by a trained radiologist and using automated software which obviated the need to perform manual measurements. Finally, we used the USDA food composition table as the Iranian one was not complete. This study also has its strength; in the current study, we adjusted the puberty status of participants for controlling the effect of hormonal changes of this period on vascular structure. Moreover, a validated and reliable FFQ was used, which was completed by expert nutritionists to minimize potential measurement errors.

## Conclusion

In conclusion we found that consumption of nuts has a desirable association with cIMT among children and adolescents with overweight and obesity. Besides, substitution of nuts for sweet unhealthy snacks could also decrease the cIMT. Our findings emphasize the favorable impact of nut consumptions as a healthy snack on subclinical atherosclerosis. Future clinical trial studies could examine the effect of different kinds of nut consumptions on cIMT and other CVD risk factors.

## Additional file

Additional file 1: **Table S1.** Standardized coefficients of snack consumption with cIMT in children and adolescents. **Table S2.** Multivariable-adjusted ORs (95% CIs) for the prevalence of high cIMT by tertiles of snack consumption in children and adolescents. (DOCX 15 kb)

## Abbreviations

AEPC: European Pediatric Cardiology
BMI: Body mass index
cIMT: Carotid intima-media thickness
CIs: Confidence intervals
CVD: Cardio vascular disease
CVs: Coefficients of variation
DBP: Diastolic blood pressure
FCT: Food composition tables
FFQ: Food frequency questionnaire
FPG: Fasting plasma glucose
MAQ: Modifiable Activity Questionnaire
MUFA: Monounsaturated fatty acid
NHANES: National Health and Nutrition Examination Survey
OR: Odds ratio
PUFA: Polyunsaturated fatty acid
SBP: Systolic blood pressure
SFA: Saturated fatty acid
TGs: Serum triglycerides
TLGS: Tehran Lipid Glucose Study
USDA: US Department of Agriculture
WHO: World Health Organization
