# Supplementary material for: Association of nuts and unhealthy snacks with subclinical atherosclerosis among children and adolescents with overweight and obesity
Source: Nutr Metab (Lond). 2019 Apr 8;16:23. doi: 10.1186/s12986-019-0350-y (PMC6454780; doi:10.1186/s12986-019-0350-y)
Supplement: Supplementary file 1 — Table S1. Standardized coefficients of snack consumption with cIMT in children and adolescents. Table S2. Multivariable-adjusted ORs (95% CIs) for the prevalence of high cIMT by tertiles of snack consumption in children and adolescents. (DOCX 15 kb) [file 12986_2019_350_MOESM1_ESM.docx]

Table S1. Standardized coefficients of snack consumption with cIMT in children and adolescents

|  | Carotid intima-media thickness | |
| --- | --- | --- |
|  | β | P value |
| Nuts (serving/wk/1000Kcal) |  |  |
| Model1 | -0.106 | 0.051 |
| Model2 | -0.135 | 0.009 |
| Model3 | -0.118 | 0.029 |
| Energy-dense nutrient-poor solid snacks (serving/wk/1000Kcal) |  |  |
| Model1 | -0.057 | 0.293 |
| Model2 | -0.008 | 0.882 |
| Model3 | -0.002 | 0.977 |

Model 1 was crude.

Model 2 was adjusted for sex, age, energy intake, pubertal status, physical activity and BMI.

Model3 was adjusted for model 2 variables plus systolic and diastolic blood pressure, SAFA, MUFA and PUFA

Linear regression was used.

Table S2. Multivariable-adjusted ORs (95% CIs) for the prevalence of high cIMT by tertiles of snack consumption in children and adolescents

|  | Intakes | | | P for trend |
| --- | --- | --- | --- | --- |
|  | T1 (n=112) | T2 (n=114) | T3 (n=113) |  |
| Median nuts (serving/wk/1000Kcal) | 0.34 | 0.99 | 2.78 |  |
| Model1 | 1.00 | 0.83(0.47-1.46) | 0.49(0.27-0.89) | 0.018 |
| Model2 | 1.00 | 0.70(0.38-1.28) | 0.41(0.21-0.79) | 0.010 |
| Model3 | 1.00 | 0.8(0.37-1.25) | 0.40(0.20-0.79) | 0.010 |
| Median energy-dense nutrient-poor solid snacks (serving/wk/1000Kcal) | 2.13 | 3.83 | 5.83 |  |
| Model1 | 1.00 | 0.95(0.54-1.69) | 0.76(0.42-1.37) | 0.367 |
| Model2 | 1.00 | 1.13(0.61-2.11) | 1.00(0.53-1.89) | 0.985 |
| Model3 | 1.00 | 1.13(0.60-2.12) | 1.01(0.53-1.92) | 0.969 |

cIMT, carotid intima-media thickness.

Model 1 was crude.

Model 2 was adjusted for sex, age, energy intake, pubertal status, physical activity and BMI.

Model3 was adjusted for model 3 variables plus systolic and diastolic blood pressure, SAFA, MUFA and PUFA

Using the median values for tertiles of snacks as continuous variables.
